# The natural alkaloid Jerantinine B has activity in acute myeloid leukemia cells through a mechanism involving c-Jun

**DOI:** 10.1186/s12885-020-07119-2

**Published:** 2020-07-07

**Authors:** Hayaa Moeed Alhuthali, Tracey D. Bradshaw, Kuan-Hon Lim, Toh-Seok Kam, Claire H. Seedhouse

**Affiliations:** 1 Blood Cancer and Stem Cells, Division of Cancer and Stem Cells, School of Medicine, Nottingham Biodiscovery Institute, University of Nottingham, Room B209, University Park, Nottingham, NG7 2RD UK; 2 College of Applied Medical Science, Taif University, Ta’if, Saudi Arabia; 3 School of Pharmacy, University of Nottingham, Nottingham, UK; 4 School of Pharmacy, University of Nottingham, Semenyih, Malaysia; 5 Department of Chemistry, University of Malaya, Kuala Lumpur, Malaysia

**Keywords:** Acute myeloid leukemia, Jerantinine, c-Jun, reactive oxygen species

## Abstract

**Background:**

Acute myeloid leukemia (AML) is a heterogenous hematological malignancy with poor long-term survival. New drugs which improve the outcome of AML patients are urgently required. In this work, the activity and mechanism of action of the cytotoxic indole alkaloid Jerantinine B (JB), was examined in AML cells.

**Methods:**

We used a combination of proliferation and apoptosis assays to assess the effect of JB on AML cell lines and patient samples, with BH3 profiling being performed to identify early effects of the drug (4 h). Phosphokinase arrays were adopted to identify potential driver proteins in the cellular response to JB, the results of which were confirmed and extended using western blotting and inhibitor assays and measuring levels of reactive oxygen species.

**Results:**

AML cell growth was significantly impaired following JB exposure in a dose-dependent manner; potent colony inhibition of primary patient cells was also observed. An apoptotic mode of death was demonstrated using Annexin V and upregulation of apoptotic biomarkers (active caspase 3 and cleaved PARP). Using BH3 profiling, JB was shown to prime cells to apoptosis at an early time point (4 h) and phospho-kinase arrays demonstrated this to be associated with a strong upregulation and activation of both total and phosphorylated c-Jun (S63). The mechanism of c-Jun activation was probed and significant induction of reactive oxygen species (ROS) was demonstrated which resulted in an increase in the DNA damage response marker γH2AX. This was further verified by the loss of JB-induced C-Jun activation and maintenance of cell viability when using the ROS scavenger N-acetyl-L-cysteine (NAC).

**Conclusions:**

This work provides the first evidence of cytotoxicity of JB against AML cells and identifies ROS-induced c-Jun activation as the major mechanism of action.

## Background

Acute myeloid leukemia is an aggressive heterogeneous clonal disorder of hematopoietic stem cells. It is characterized by defects in the self-renewal and differentiation programs that regulate myeloid cell production causing accumulation of immature, non-functional cells termed myeloblasts. Despite advances in the outcome of younger AML patients, long-term remission is still not achieved in the majority of cases and AML in the elderly, which is often correlated with adverse risk factors, is associated with poor clinical outcome [1]. Whilst initial clinical results in patients treated with small molecule inhibitors are promising, relapse often occurs due to the emergence of acquired resistance mechanisms; there therefore remains an unmet need for drugs acting on other signaling pathways.

Natural products represent important sources of drugs and drug-scaffolds, and natural product-inspired therapies continue to have significant impact in the cancer arena [2]. Work by Lim et al. [3] on the leaf extracts of the Malayan plant *Tabernaemontana corymbosa* resulted in the isolation and purification of a series of new alkaloids, the Jerantinines, which have demonstrated promising biological activity. The majority of published work has been on Jerantinines A and B (JA and JB), reporting in vitro antitumor activities of these agents against various solid human-derived carcinomas. Specifically, JA and JB have been shown to inhibit the growth and colony formation of cancer cell lines accompanied by induction of apoptosis in a dose- and time-dependent manner [4, 5]. JA and JB potently inhibited tubulin polymerization and caused severe perturbation of microtubule dynamicity [4, 5]. X-ray crystallography studies demonstrated the colchicine site as the binding site of JB acetate (JBa) on microtubules [6].

JA and JB were also found to inhibit the activity of kinases involved in mitosis and significantly evoke potent G2/M cell cycle arrest with PLK1 being targeted in a dose-dependent manner [5]. An additional mechanism of action in non-hematological cancers included modulation of splicing [7].

These findings encouraged us to assess JB activity in AML cells, with the aims of establishing whether this natural product would provide potential effective targeting of AML and to elucidate the main mechanism of drug action in AML cells.

## Methods

### Materials

10 mM stocks of JB and JBa were stored in dimethyl sulphoxide (DMSO) at − 80 °C protected from light. Unless otherwise stated IC_50_ JB concentrations were used.

### AML cell lines and primary samples

MV4–11 and HL-60 myeloid leukemia cell lines were grown in Roswell Park Memorial Institute (RPMI-1640) medium supplemented with 10% fetal calf serum (FCS: 02–00-850; First Link), 2 mM L-glutamine (G7513, Sigma), 10 μg/ml streptomycin and 100 U/ml penicillin. KG-1a cell line was cultured as above but supplemented with 20% FCS. MV4–11 was purchased from the American Tissue Culture Collection (Manassas, USA). HL-60 and KG1a were purchased from the European Collection of Animal Cell Culture (Salisbury, UK). All cells were incubated at 37 °C in 5% CO_2_ and assays were set up using cells in the log phase of growth. Continued testing to authenticate these cell lines was performed using multiplex short tandem repeat analysis (Powerplex 16, Promega) and mycoplasma testing was carried out routinely using the Mycoalert mycoplasma detection kit (Lonza).

Blood or bone marrow samples were obtained from AML patients presenting to Nottingham University Hospital following informed consent. Mononuclear cells were isolated from AML patient samples using a standard density gradient/centrifugation method and clonogenic assays were carried as previously described using 2 × 10^4^ cells per well. Growth was defined by the presence of > 12 colonies in untreated conditions [8].

### Cell viability assays

Cell viability was initially assessed using Alamar Blue (AbD Serotec) according to the manufacturer’s instructions. Cell counting using a hemocytometer was also undertaken.

Apoptosis was examined using the Annexin V-FITC apoptosis detection kit (Trevigen) according to manufacturer’s instructions. Cleaved PARP was measured in cells fixed in 4% paraformaldehyde using Alexa Fluor 647 Conjugate (BD Biosciences). Analyzes were performed by flow cytometry using a FACS Canto II (BD Biosciences). Assessment of activated caspase was made on cells fixed and permeabilized using a Leucoperm kit (AbD Serotec), active caspase 3 was measured using PE-conjugated polyclonal rabbit anti-active caspase-3 (BD Pharmingen).

### Dynamic BH3 profiling

Cells at 5 × 10^5^/ml were incubated with the IC_50_ concentration of JB in culture medium for 4 h. Cytochrome C release was measured as previously described. Adjustments for peptide induced cytochrome C release in untreated cells were made in order to establish agent-specific release, using the formula 100*(release with agent – release without agent)/(100 – release without agent) [9].

### Identification of target proteins

A Proteome Profiler Human Phospho-Array (R&D Systems) was used to analyze the phosphorylation profile in cells according to the manufacturer’s instructions. Results were confirmed using western blot analysis with anti-rabbit total c-Jun (Abcam 32137), anti-rabbit phospho c-Jun (S63) (Abcam 32385) and loading control mouse anti-Lamin (Santa Cruz # SC-7292). C-jun was probed for first, followed by membrane striping and probing for lamin.

### Determination of intracellular ROS

Cells at a density of 5 × 10^5^/ml medium were treated with JB and incubated at 37 °C for 4 h. Twenty-five mins prior to the end of incubation, 3 μM chloromethyl dihydro 2′7’dichlorofluorescein diacetate (CM-H2DCFDA) (Invitrogen) was added to cells. At the completion of incubation, samples were placed on ice and the fluorescent oxidation product measured immediately by FACS Canto II flow cytometry. N-Acetyl-L-Cysteine (NAC) and SP600125 JNK inhibitor (JNKI) were purchased from Sigma (A7250) and Abcam (ab120065) respectively. Further dilutions were made in cell culture medium.

### Assessment of DNA damage response (DDR) marker (H2AX Ser139)

H2AX phosphorylation on Ser139 (γH2AX) was examined by flow cytometry with a kit from Upstate (Millipore cat# 16–202) according to the manufacturer’s instructions.

### Statistical analysis

Statistical analyses were performed using paired T-test. Significance was defined as a *p* < 0.05.

## Results

### Jerantinine B inhibits the growth of AML cells in a dose-dependent manner

**Fig. 1:**
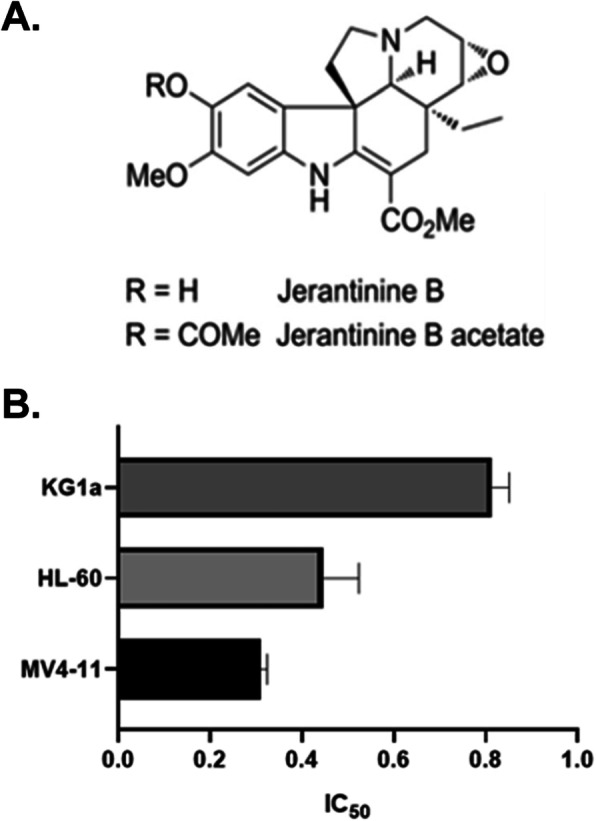
*Cytotoxicity of jerantinine B in AML cell lines.***a**. Structure of JB and JBa. **b**. Mean IC_50_ values of JB at 24 h. Columns, mean of three independent experiments; bars, SD

The structure of JB is shown in Fig. 1a. IC_50_ values were determined at 24 h exposure to JB for cell lines using alamar blue assay and cell counting. The cell lines demonstrated similar sensitivities with IC_50_ values: MV4–11 0.3 μM; HL-60 0.4 μM and KG1a 0.8 μM (Fig. 1b). Due to the comparable drug sensitivities, further assays were not always performed on all three of the cell lines.

**Fig. 2:**
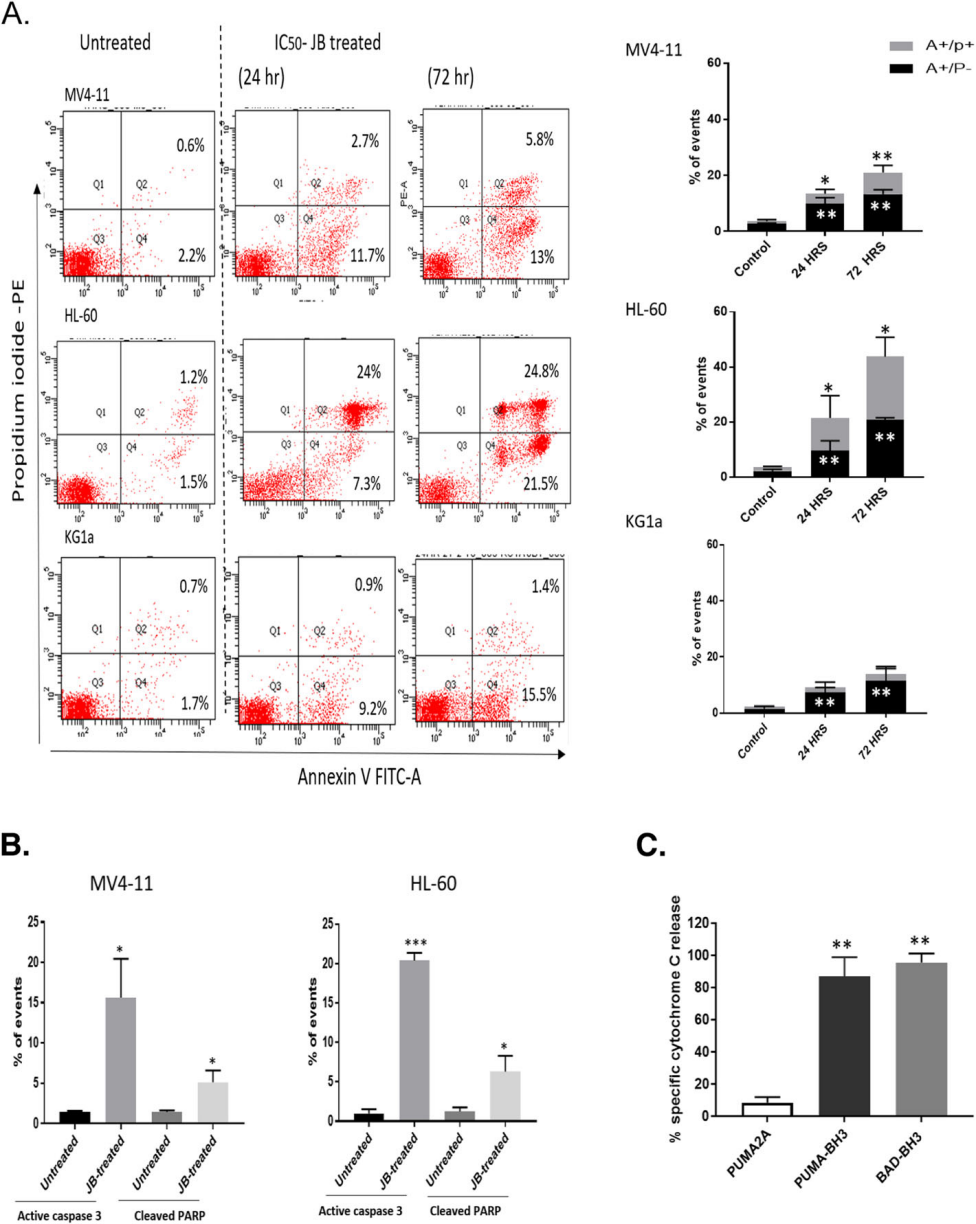
*Induction of apoptosis in IC*_*50*_*JB exposed AML cells.***a**. Flow cytometric analysis of Annexin V/propidium iodide staining following IC_50_-JB treatment for 24 and 72 h. Representative flow cytometry plots and summary histograms are shown. A+/PI− indicates cells undergoing early stage apoptosis, while A+/PI+ defines late stage apoptotic populations. **b**. Summary bar chart of flow cytometric analysis of cleaved PARP and active caspase 3 apoptotic markers following 24 h IC_50_ JB exposure. **c**. BH3 profiling following 4 h IC_50_-JB treatment in MV4–11 cells. Cytochrome C release demonstrates PUMA- and BAD-BH3 peptides prime the cells to apoptosis. PUMA2A is a mutated peptide which acts as a negative control . Columns, mean of at least three independent experiments; bars, SD. * *P* < 0.05, ** *P* < 0.01, *** *P* < 0.001

Annexin V assays were performed to establish whether JB induced apoptosis after 24 and 72 h exposure. Figure 2a demonstrates significant apoptotic cell death resulting from IC_50_-JB treatment in all cell lines when compared to untreated controls (*p* < 0.05) in a time-dependent manner. This was particularly profound in the HL-60 cell line at 72 h.

Apoptotic markers were also assessed to further confirm apoptotic cell death in JB-exposed cells. Using MV4–11 and HL-60 cells treated with IC_50_ JB, active caspase 3 and cleaved PARP apoptotic markers were shown to increase significantly (*p* < 0.05) when compared to untreated controls (Fig. 2b).

JB was shown to affect the cell cycle in AML cell lines and cause transient G2/M arrest, however the increase was not as profound as in solid cancer cell lines (additional file 1).

### JB exerts an early apoptotic effect on AML cells

BH3 profiling assays on MV4–11 cells demonstrated that JB has an early effect with cells being primed for apoptosis within 4 h. Figure 2c shows that when using the negative control, mutated PUMA2A peptide, there is no induction of cytochrome C release, indicating that JB alone does not induce cytochrome C (and thus apoptosis) at this time point. However, when PUMA-BH3 or BAD-BH3 are added, cytochrome C release occurs indicating that JB has primed the cells to undergo apoptosis.

### JB induces c-Jun activation in leukemia cell lines

**Fig. 3:**
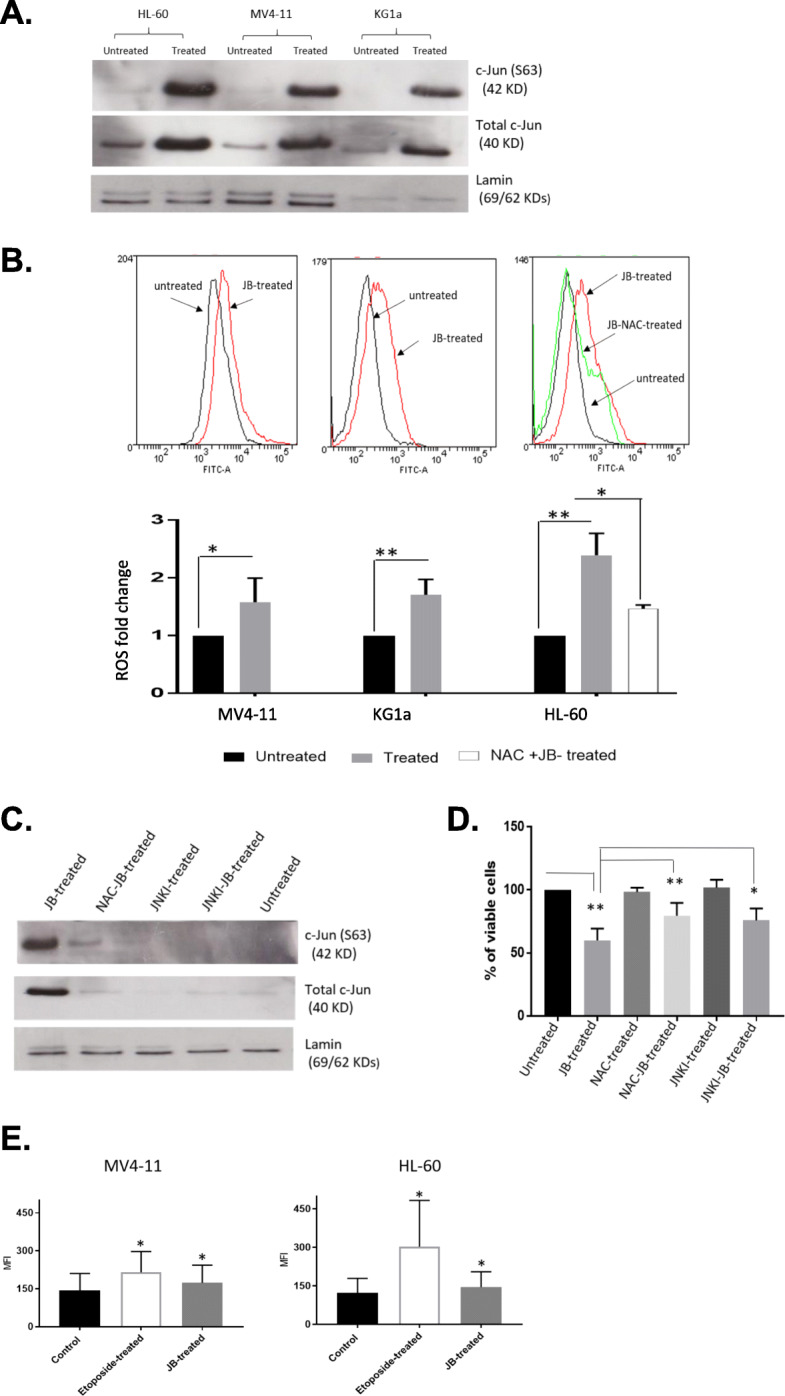
*JB activates c-Jun through ROS induction.***a**. Western blot (cropped) demonstrating 4 h JB exposure results in a strong upregulation of total c-Jun and activation of c-Jun (S63 phosphorylation) in AML cell lines. Lamin is shown as the loading control and the figure is representative of three independent experiments. **b**. JB induced intracellular ROS in AML cell lines. The bar charts indicate the fold change in median fluorescence intensity compared to untreated controls upon addition of the oxidative stress indicator CM-H2DCFDA, with the elimination of ROS seen when the anti-oxidant NAC is included. Representative flow cytometry plots of ROS measurements are shown above the corresponding bar charts. **c**. Western blot results (cropped) showing elimination of JB-dependent c-Jun activation by either ROS scavenger or JNKI, representative of three independent experiments. **d**. cell counts after 24 h incubation showing a combination of ROS scavenger or JNKI with JB treatment reversed JB-induced cell death, displayed as % viability of untreated control. **e**. DNA damage, assessed by the response marker γH2AX, is increased in JB-treated cells. Bars represent the mean of the Median Fluorescence Intensity (MFI) in respect to the negative untreated control. Etoposide was used as a positive control*.* Columns, mean of at least three independent experiments; bars, SD. * *P* < 0.05 and ** *P* < 0.01*.* Full length blots for the westerns in this figure are shown in additional file 3

A protein kinase array was used to identify changes at the 4 h time point with prominent phosphorylation seen in the c-Jun/JNK signaling pathway. JB-treated MV4–11 and HL-60 cells exhibited a high level of phosphorylation in JNK1/2/3 and c-Jun S63 compared to the untreated samples (additional file 2). Western blotting confirmed increased levels of total and phosphorylated (S63) c-Jun after 4 h JB exposure. Figure 3a shows that 4 h exposure to JB resulted in strong expression of total and phosphorylated c-Jun protein in all cell lines studied.

### JB induces reactive oxygen species (ROS)

c-Jun/JNK has previously reported to be activated in cells exposed to oxidative stress [10, 11]. ROS levels in JB treated cells were therefore determined at 4 h using oxidative stress indicator CM-H2DCFDA. In comparison to the control group, IC_50_ JB treatment produced significantly increased ROS levels (Fig. 3b). Flow cytometric analysis demonstrated that ROS levels were increased by 2.39 (*P* = 0.002), 1.57 (*P* = 0.03) and 1.70-fold (*P* = 0.006) in HL-60, MV4–11 and KG1a respectively. Confirmatory assays with HL-60 cells demonstrated that co-treatment of cells with JB and the antioxidant NAC abolished JB-induced ROS (P = 0.03) (Fig. 3b).

### Association between ROS generation and c-Jun/JNK activation in JB-induced AML cell death

Upon establishing that JB generated significant levels of ROS, we aimed to establish whether scavenging of ROS resulted in the inhibition of c-Jun activation following JB-treatment. A JNK inhibitor (JNKI) was used as a positive control. HL-60 cells were pre-incubated with 20 μM JNKI for 1 h to allow JNK inhibition and then treated with JB for up to 24 h. Lysates were prepared after 4 h of incubation for assessing c-Jun protein expression and cell counting was performed after 24 h for evaluating cell viability. Immunoblotting results revealed that co-treatment with JB and the antioxidant NAC significantly reduced JB-induced c-Jun activation to levels comparable with the JNKI- JB co-treated sample (Fig. 3c). These data support the involvement of ROS in JB activity. Subsequently, a cell viability assay demonstrated that JB-treated samples exhibited 59.93% ± 9.38 viability that was increased significantly in JB-NAC and JNKI-JB treated samples (*P* = 0.003 and 0.02 respectively) (Fig. 3d). This suggests that JB-mediated intracellular oxidative stress acts as a signal for c-Jun/JNK-induced death in AML cells.

### DNA damage assessment

Establishing significant generation of ROS by JB led us to evaluate the potential of JB to cause DNA damage. This was investigated by flow cytometric detection of the DNA damage response protein, gamma-H2AX (γH2AX). Figure 3e represents the measurement of γH2AX after 4 h exposure to JB in MV4–11 and HL-60 showing that γH2AX was significantly increased in both cell lines (*P* < 0.05). Etoposide, a known inducer of DNA DSBs, was used as a positive control.

### JB acetate (JBa) inhibits colony formation of primary AML patient cells

**Fig. 4:**
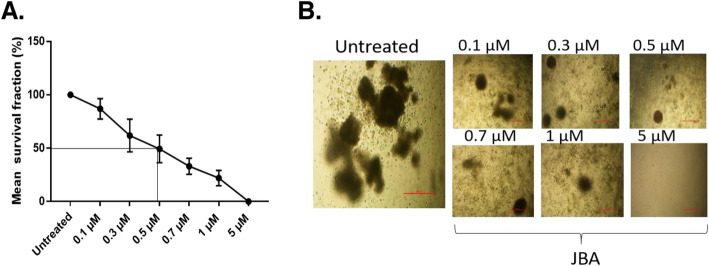
*Effect of JB on colony formation of AML primary cells.***a**. Survival fraction of four AML patient samples when treated with JB. Results are displayed as mean ± SD of survival fraction percent. **b**. Representative images of AML cells from a patient sample showing the effect of JBA on colony formation at a range of drug concentration

To demonstrate that JB is also effective in primary AML cells, JB acetate (JBa) was tested on four patient samples in clonogenic assays. For this long-term assay, the acetate derivative of JB was used; as it demonstrates increased stability and reduction of overall polarity [3]. Fresh diagnostic AML samples were grown for 14 days in a methylcellulose-based medium containing 0 to 5 μM JBa. All samples exhibited sensitivity to JBa and with IC_50_ values 0.47 ± 0.11 (Fig. 4).

## Discussion

In this study, cytotoxicity assays established that JB exhibited potent anti-proliferative activities against AML cell lines accompanied by time- dependent apoptotic cell death. JBa, an acetate derivative of JB, resulted in a dose- dependent inhibition of colony formation in primary AML cells indicating cell death or loss of capacity to divide and form progeny colonies. JBa may act as a prodrug with its bio-activation requiring the presence of cellular esterases [12]. As clonogenic assays are performed with a low density of cells, with subsequent low esterase activity, there may be a low bioavailability of the test agent [12], meaning the effects of JBa on AML patient primary cells was potentially underestimated. Importantly, the concentrations used in this work have been demonstrated to be pharmacologically achievable [5].

Results of BH3 profiling assays on MV4–11 cells suggested that JB has an early effect with cells being primed to undergo apoptosis by 4 h. Thus, we examined changes in phosphorylation in an array of protein kinases to identify changes at this time point. This investigation revealed the activation of mitogen-activated protein kinases (MAPKs) in JB-treated cell lines. It specifically established that JB caused strong activation of c-Jun/JNK signaling.

It has been long established that many natural products have pro-oxidant properties [13–15]. The JNK pathway is one of the major signaling cascades of the MAPK signaling pathway that is activated when cells have been exposed to various forms of environmental stress stimuli including ROS. Mitochondrial release of ROS was found to cause JNK activation [11, 16]. In the current study, we have established the activation of c-Jun/JNK by JB in AML cells through ROS induction. Pharmacological inhibition of JNK confirmed the requirement of activated c-Jun/JNK for JB- induced apoptosis. These findings indicate that the effect of JB in AML cells is dependent on oxidative stress that acts as an early trigger for c-Jun activation, and we suggest that the main molecular targets are via c-Jun/JNK signaling. Indeed, numerous agents with pro-oxidant properties have been shown to be effective against both primary leukemic blasts and leukemic cell lines. It has been reported that clinically achievable concentrations of arsenic trioxide, an agent approved for treating APL has pro-oxidant capacity and mediates apoptosis through three mechanisms: increasing endogenous ROS production, activating MAPKs and also activating caspases in the leukemic cells [17]. In addition, it has been demonstrated that quercetin, a plant-derived bioflavonoid, enhances the production of intracellular oxidative stress causing mitochondrial membrane depolarization, cytochrome C release, sustained activation of ERK and ultimately induction of apoptosis in HL-60 cells in vivo and in vitro [18]. More recently, work has shown that tricetin, a dietary flavonoid in Myrtaceae pollen and eucalyptus honey, induces a JNK- induced apoptosis pathway in the HL-60 cell line by enhancing ROS generation, and co-treatment with the ROS scavenger, NAC, abolished tricetin- mediated JNK activation and subsequent cell apoptosis [11].

Increased levels of ROS have previously been shown to perturb cell cycle dynamics, causing G2 phase arrest [19] and correlate with increased DNA damage. In solid cancer models, JB caused significant G2/M cell cycle arrest accompanied by generation of ROS and detection of γH2AX [5]. Clinically, the drug Vorinostat has also been reported to induce ROS production and cause DNA damage [20]. Consequently, establishing significant generation of ROS and cell cycle perturbation by JB led us to investigate the potential of JB to cause DNA damage. Measurement of the DDR marker (γH2AX) exhibited a significant increase in both the studied cell lines following 4 h JB exposure (*P* < 0.05).

Oxidative stress induction in hematopoietic progenitors and leukaemia cells has been reported to cause myeloid cell differentiation [21]. More recently, and of interest to this work, it has been established that increased ROS levels by phorbol-12-myristate-13-acetate (PMA), activate transcription of differentiation genes in AML cells via the c-Jun/JNK signalling pathway [22]. We have preliminary evidence to suggest that JB induces differentiation in AML cells (data not shown) and this is an avenue we will explore further in future work.

It has previously been reported that microtubule polymerization is the major molecular target affected by JA and JB in solid cancers [4, 5]. In the current study, the effect of JB on microtubules was not investigated. However, transient G2/M- and S-phase cell cycle blockade (additional file 1) is a key indicator of microtubule disruption and evidence is also available documenting the link between microtubules and the JNK pathway. JNK activity determines the fate of microtubules during their life cycle [23] and the JNK pathway was found to be activated by microtubule inhibitors in a wide variety of cell lines [24] The early phosphorylation of JNK has been reported as a specific mechanism mediating microtubule depolymerization and G2/M arrest [25]. Further work suggests that the activation of JNK is needed for, or contributes to, cell death mediated by microtubule disrupting agents [24, 26, 27].

## Conclusion

**Fig. 5:**
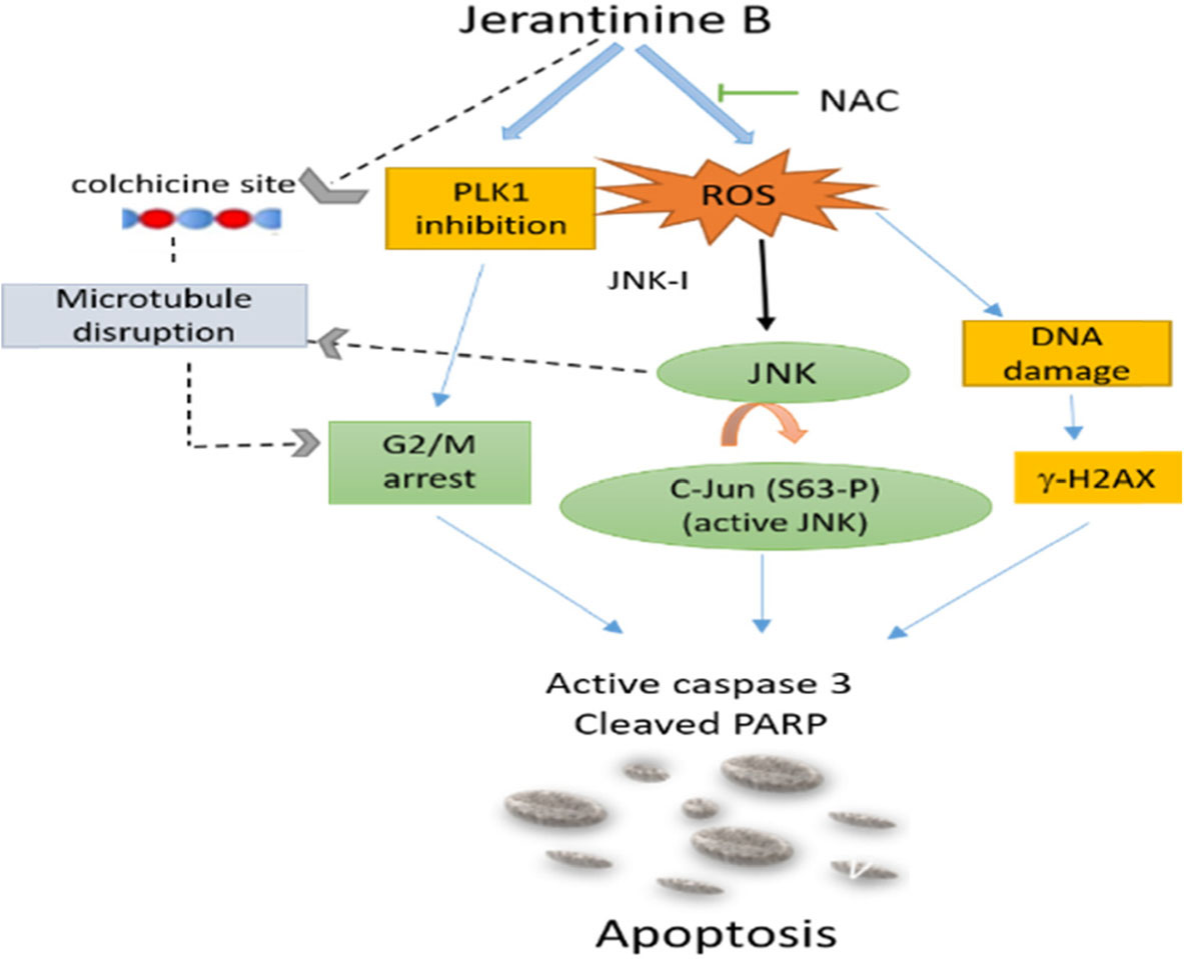
*Suggested mechanism of action of JB in AML cells.* JB in AML exerts its effect through increasing ROS level that cause c-Jun/JNK activation as well as DNA damage. JB also targets PLK1 that contributes to G2/M arrest. Activated c-Jun/JNK may contribute to microtubule disruption and ultimately G2/M arrest. JB was reported to bind directly to the colchicine site on microtubule and inhibits microtubule polymerisation but this was not tested in this study

Our investigation of this natural product provides the first evidence of cytotoxicity of JB against AML cells and elucidates the mechanism of drug action; this is schematically illustrated in Fig. 5. Thus, JB appears to be a potential chemotherapeutic agent in AML and is worthy of continued development.

## Supplementary information

**Additional file 1. **Cell cycle analysis following JB treatment. A. Summary histogram illustrating the proportion of cells in each phase of the cell cycle after 4 and 6 h treatment with IC_50_ dose of JB. B. example of flow cytometric DNA (7-AAD) content histograms (6 h-JB-treated cells). C. Cell cycle analysis after 24 h exposure to JB.D. example of flow cytometric analysis at 24 h showing reduction in BrdU-positive (dividing) cells following JB treatment. Columns, mean of three independent experiments; bars, SD. * *P* < 0.05, ** *P* < 0.01, *** *P* < 0.001.

**Additional file 2.** Phospho-kinase measurements following 4 h JB treatment. Pixel densities in A. MV4–11 and B. HL-60 JB-treated cells. Black and grey bars are untreated and treated samples respectively. C. shows the whole film image.

**Additional file 3.** Representative image of whole film of western blot. 3: Full image of western blot. A. Full western blot film image for data in Fig. 3a showing upregulation of total and active (S63 phosphorylation) c-Jun following JB treatment. The red boxes indicate where the blot was cropped for Fig. 3a. B. Full length western blot film images for data in Fig. 3c showing elimination of JB-dependent c-Jun activation by either ROS scavenger or JNKI. The red boxes indicate where the blot was cropped for Fig. 3c.

## Data Availability

All data generated or analysed during this study are included in this published article [and its supplementary information files].

## Abbreviations

AML: Acute myeloid leukemia
CM-H2DCFDA: Chloromethyl dihydro 2′7’dichlorofluorescein diacetate
DMSO: Dimethyl sulfoxide
FCS: Fetal calf serum
JA: Jerantinine A
JB: Jerantinine B
JBa: Jerantinine B acetate
MFI: Median fluorescence intensity
NAC: N-acetyl-L-cysteine
PARP: Poly (ADP-ribose) polymerase
ROS: Reactive oxygen species
SD: Standard deviation
