# Supplementary figures and images for: The natural alkaloid Jerantinine B has activity in acute myeloid leukemia cells through a mechanism involving c-Jun
Source: BMC Cancer. 2020 Jul 7;20:629. doi: 10.1186/s12885-020-07119-2 (PMC7341637; doi:10.1186/s12885-020-07119-2)

**Additional file 1**

| **A.**  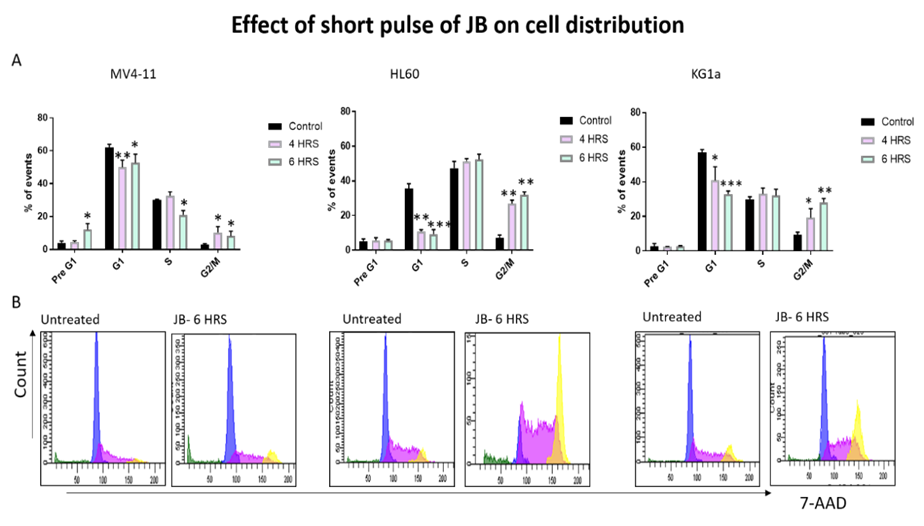  **B.**  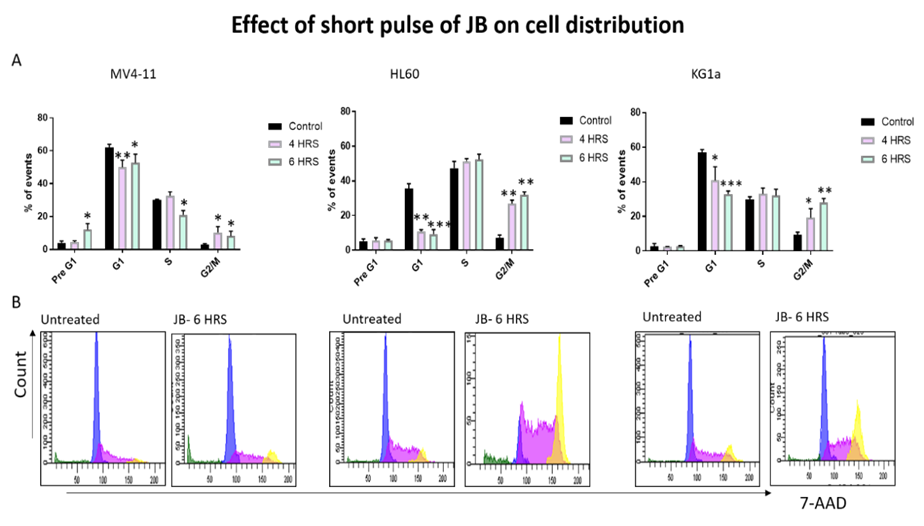  **C.**  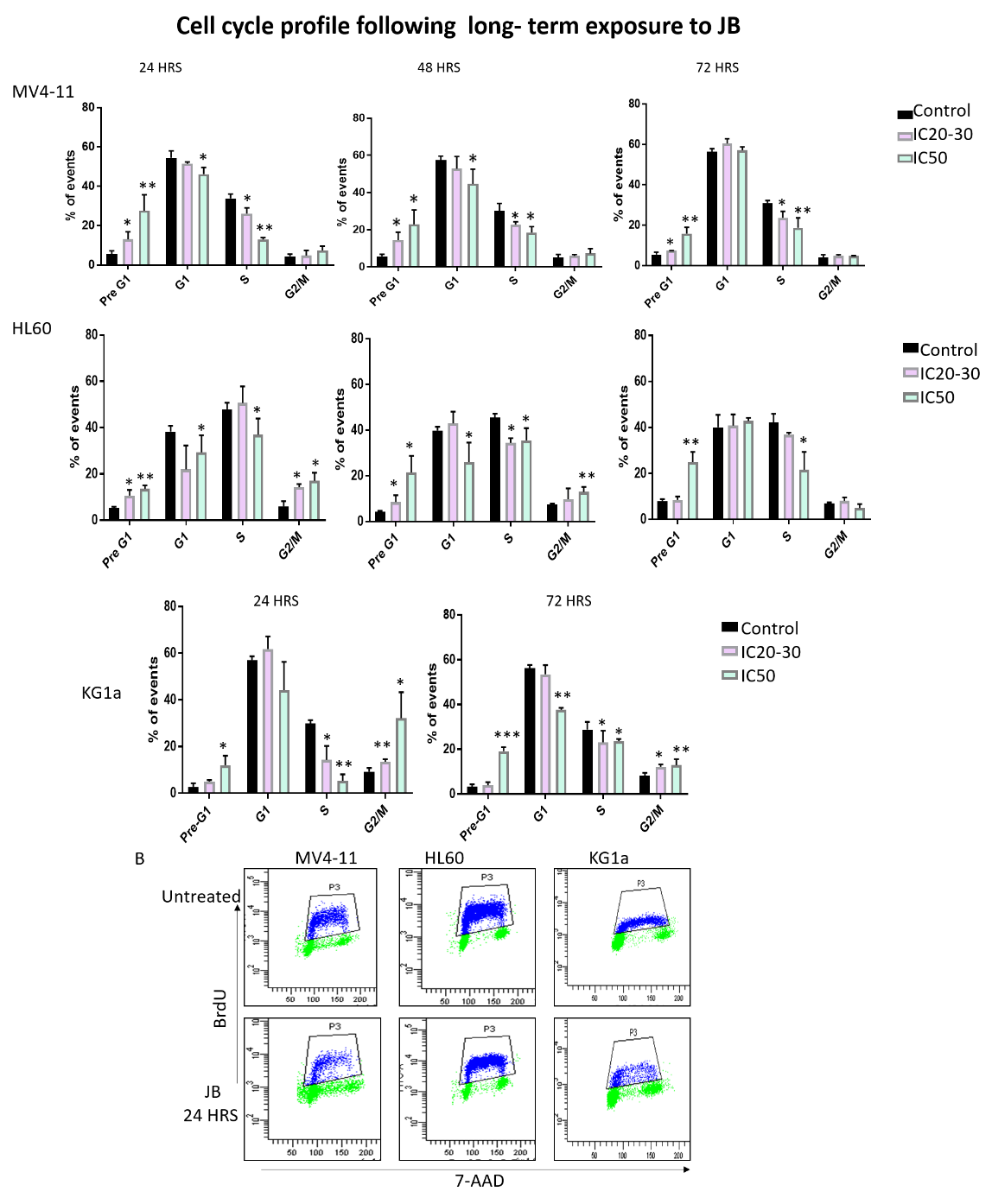 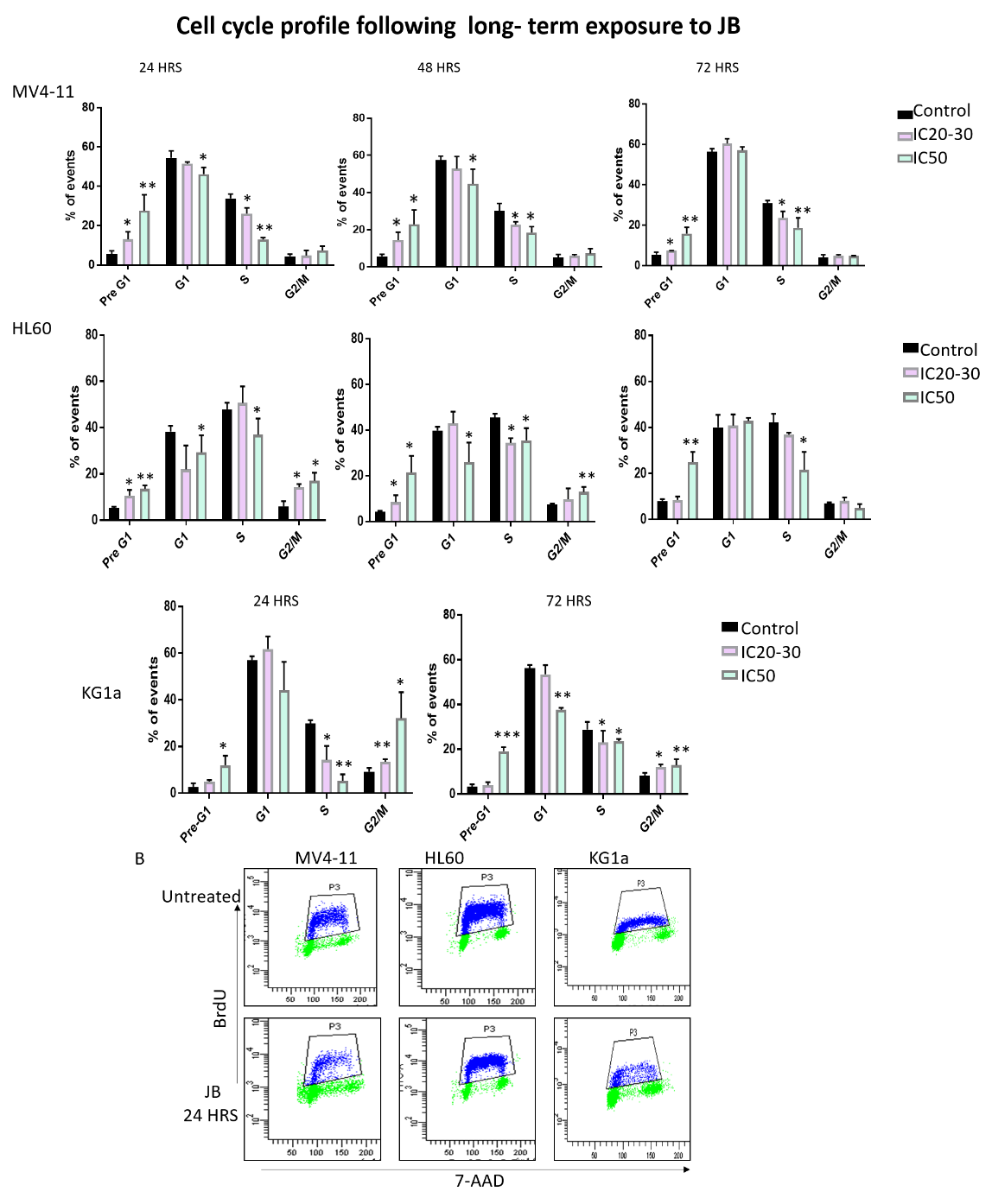 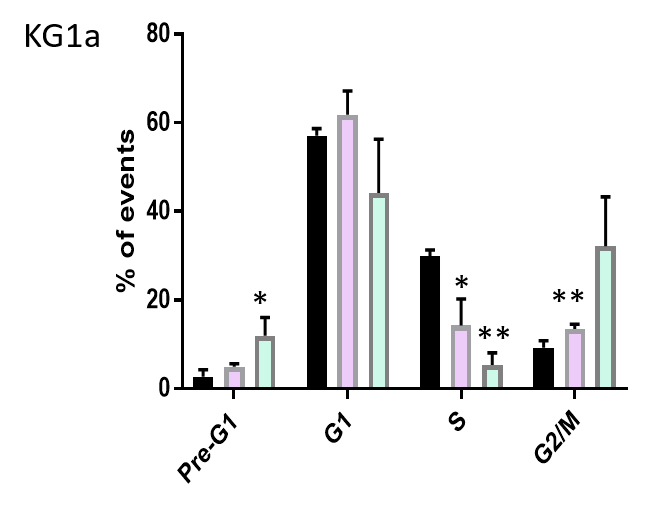  **D.**  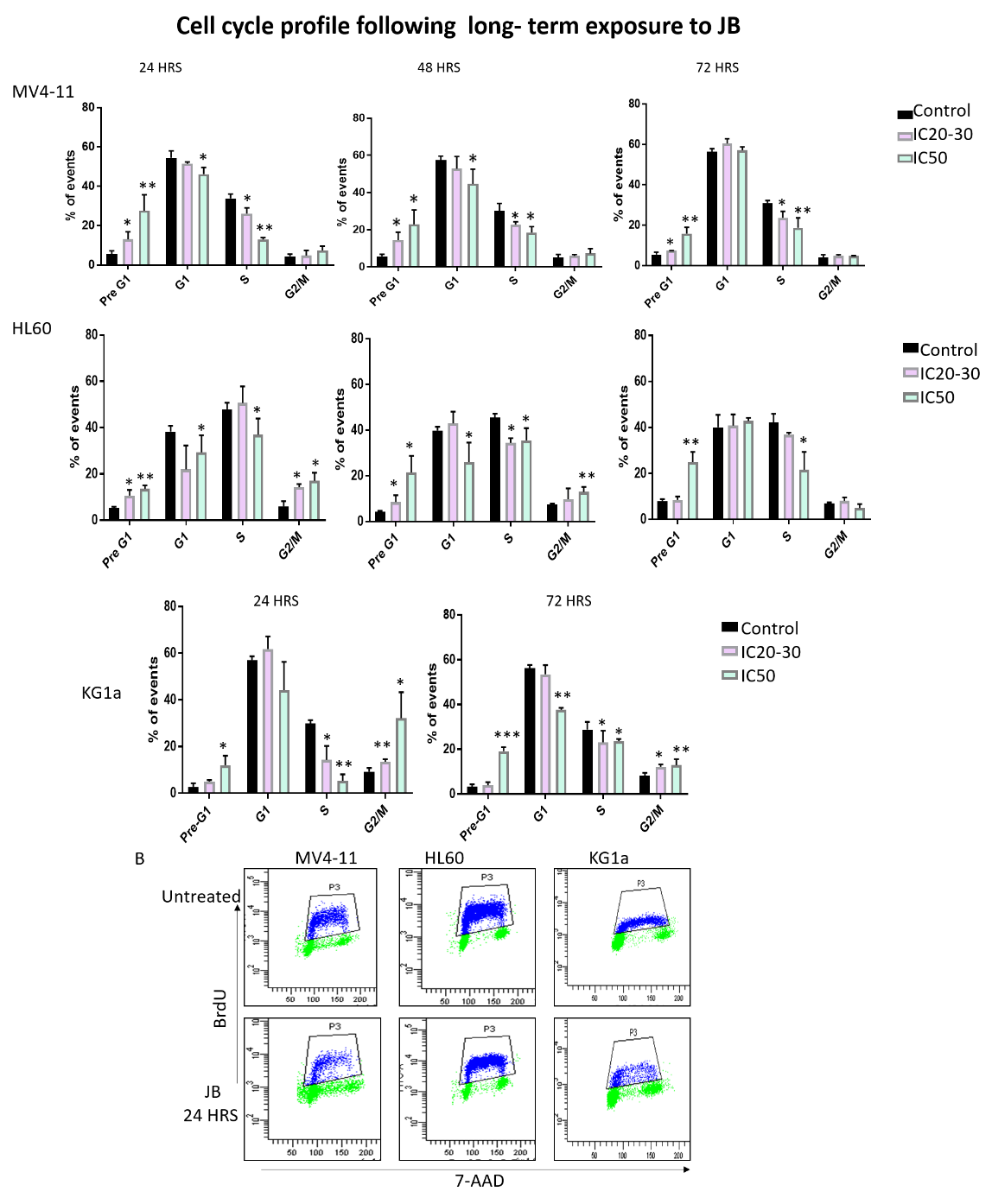 |
| --- |

Supplement: Supplementary file 1 — Additional file 1. Cell cycle analysis following JB treatment. A. Summary histogram illustrating the proportion of cells in each phase of the cell cycle after 4 and 6 h treatment with IC50 dose of JB. B. example of flow cytometric DNA (7-AAD) content histograms (6 h-JB-treated cells). C. Cell cycle analysis after 24 h exposure to JB.D. example of flow cytometric analysis at 24 h showing reduction in BrdU-positive (dividing) cells following JB treatment. Columns, mean of three independent experiments; bars, SD. * P < 0.05, ** P < 0.01, *** P < 0.001. [file 12885_2020_7119_MOESM1_ESM.docx]

**Additional file 2**

**A.**


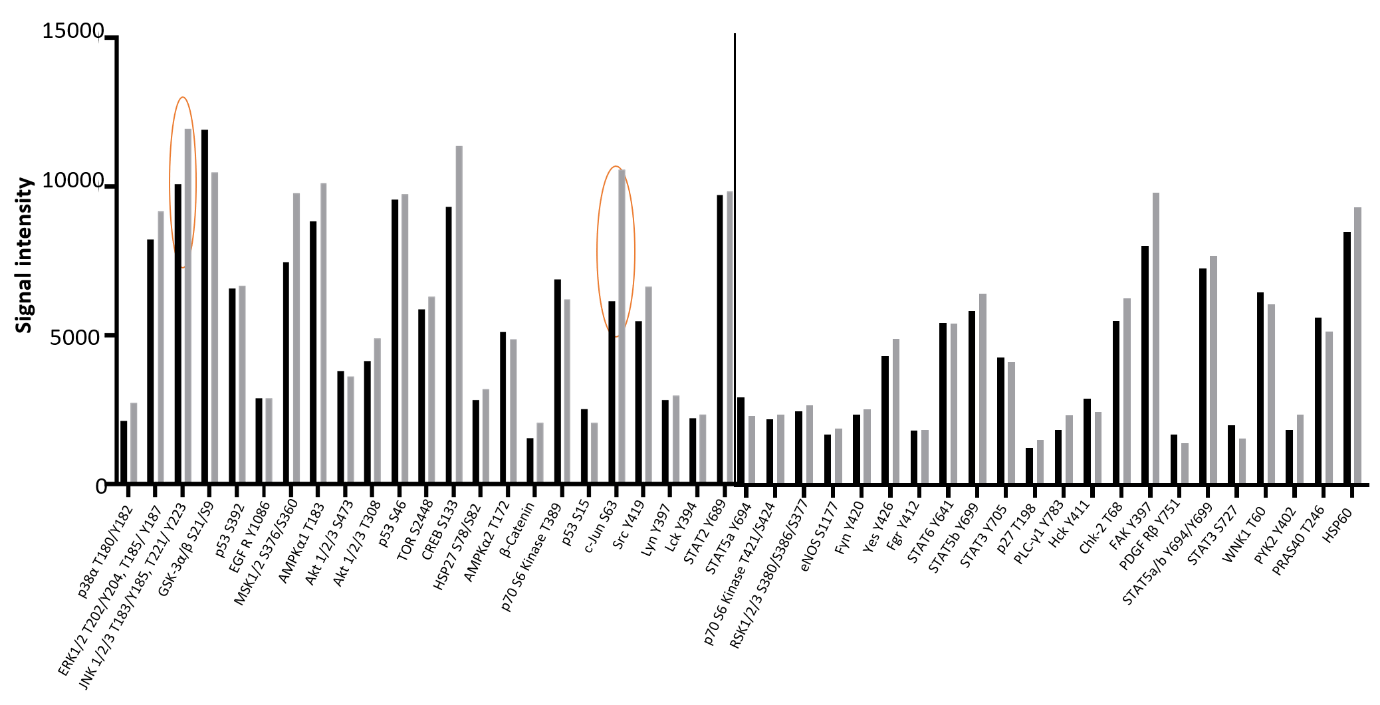


**B.**


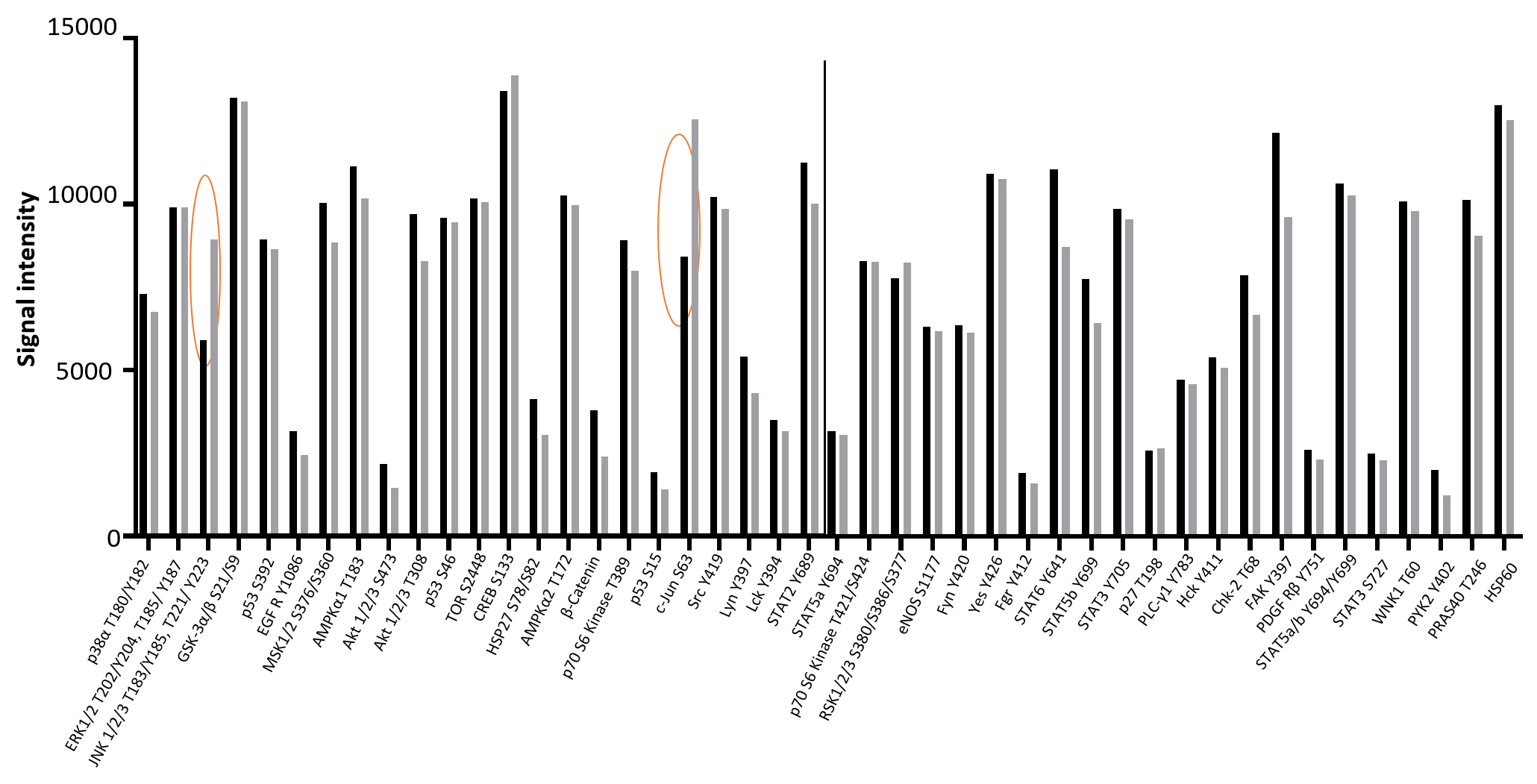


**C.**


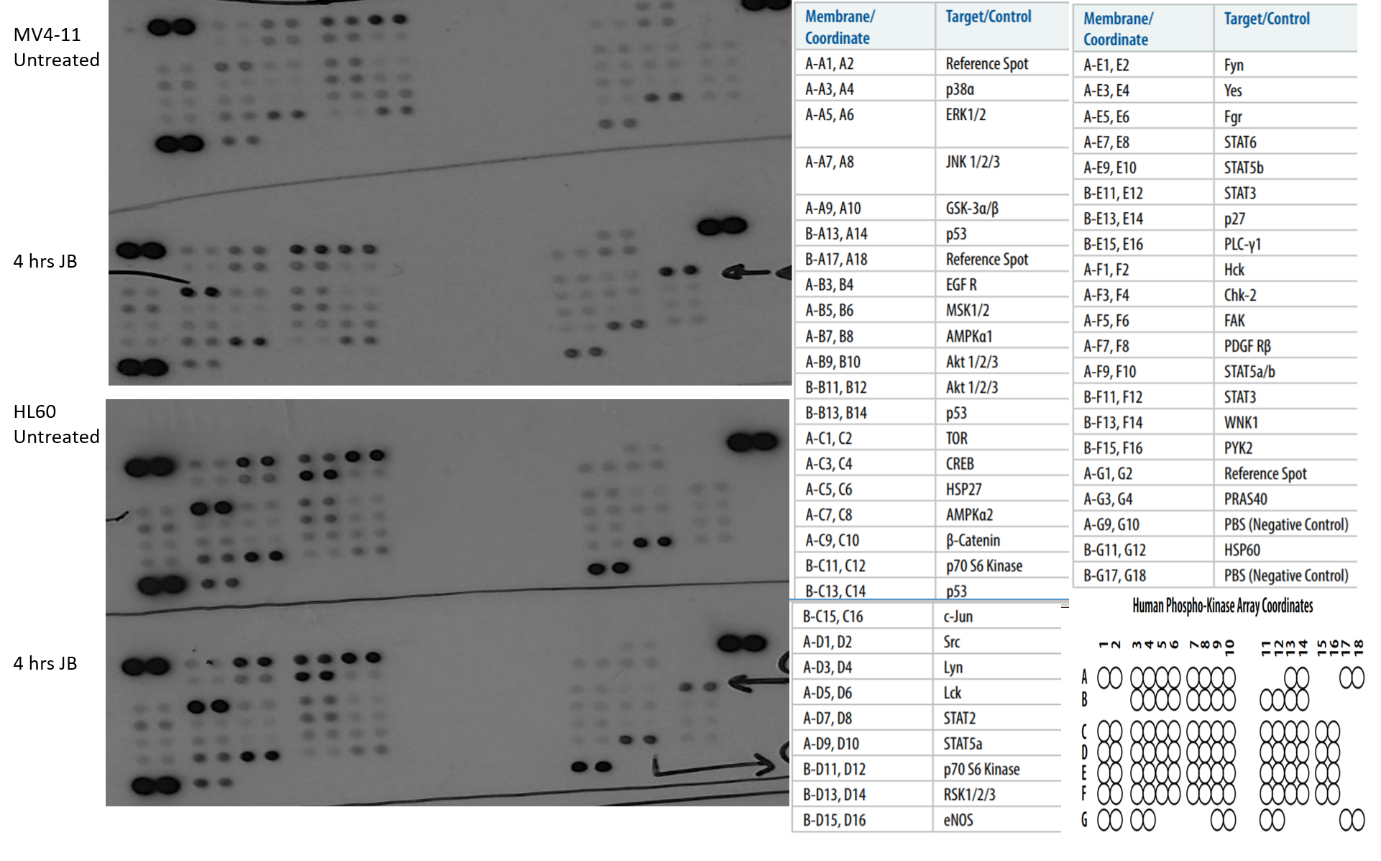

Supplement: Supplementary file 2 — Additional file 2. Phospho-kinase measurements following 4 h JB treatment. Pixel densities in A. MV4–11 and B. HL-60 JB-treated cells. Black and grey bars are untreated and treated samples respectively. C. shows the whole film image. [file 12885_2020_7119_MOESM2_ESM.docx]
