# Supplementary material for: The natural alkaloid Jerantinine B has activity in acute myeloid leukemia cells through a mechanism involving c-Jun
Source: BMC Cancer. 2020 Jul 7;20:629. doi: 10.1186/s12885-020-07119-2 (PMC7341637; doi:10.1186/s12885-020-07119-2)

**Additional file 3**

**A.**


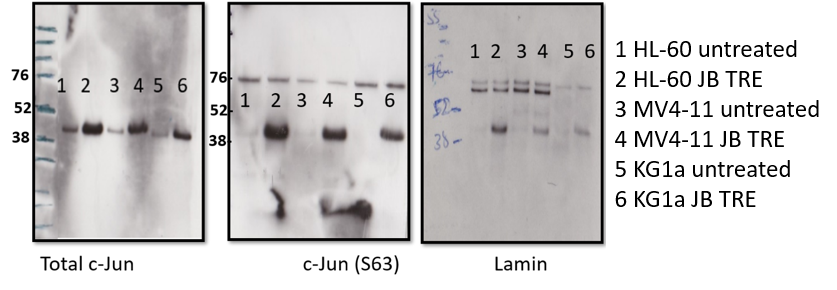


1. HL-60 untreated

2. HL-60 JB treated

3. MV4-11 untreated

4. MV4-11 JB treated

5. KG1a untreated

6. KG1a JB treated

**B.**


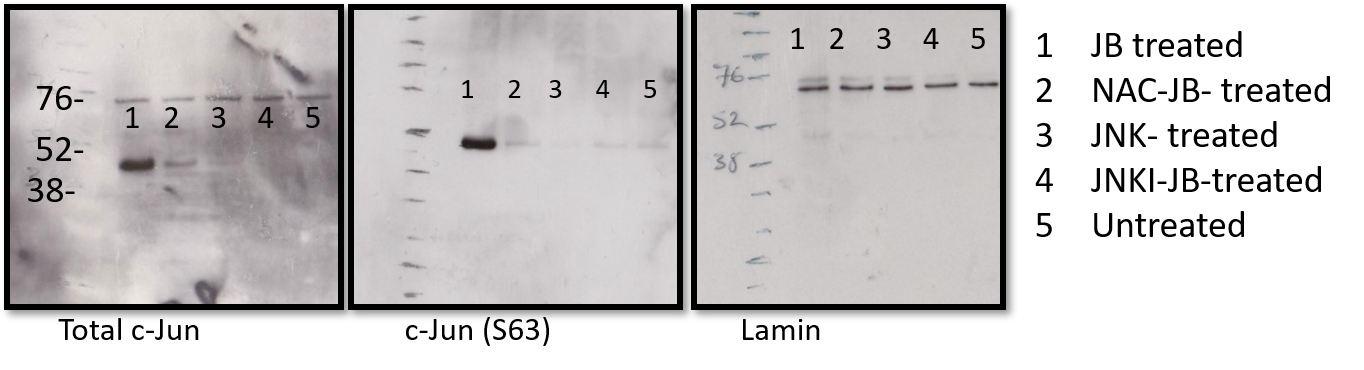

Supplement: Supplementary file 3 — Additional file 3. Representative image of whole film of western blot. 3: Full image of western blot. A. Full western blot film image for data in Fig. 3a showing upregulation of total and active (S63 phosphorylation) c-Jun following JB treatment. The red boxes indicate where the blot was cropped for Fig. 3a. B. Full length western blot film images for data in Fig. 3c showing elimination of JB-dependent c-Jun activation by either ROS scavenger or JNKI. The red boxes indicate where the blot was cropped for Fig. 3c. [file 12885_2020_7119_MOESM3_ESM.docx]
